# Mannitol versus furosemide in patients with thoracic malignancies who received cisplatin‐based chemotherapy using short hydration: A randomized phase II trial

**DOI:** 10.1002/cam4.6839

**Published:** 2024-03-08

**Authors:** Eriko Murakami, Hiroaki Akamatsu, Shunsuke Teraoka, Toshiaki Takakura, Eri Takase, Masanori Tanaka, Takahiro Kaki, Yuhei Harutani, Katsuyuki Furuta, Takeya Sugimoto, Ryota Shibaki, Daichi Fujimoto, Atsushi Hayata, Yuichi Ozawa, Masanori Nakanishi, Yasuhiro Koh, Toshio Shimokawa, Nobuyuki Yamamoto

**Affiliations:** ^1^ Internal Medicine III Wakayama Medical University Wakayama Japan; ^2^ Center for Biomedical Sciences Wakayama Medical University Wakayama Japan; ^3^ Clinical Study Support Center Wakayama Medical University Wakayama Japan

**Keywords:** chemotherapy, clinical cancer research, clinical trials, lung cancer, quality of life

## Abstract

**Background:**

Mannitol is exclusively recommended in the National Comprehensive Cancer Network guidelines for diuresis in cisplatin (CDDP)‐based chemotherapy. The utility of furosemide, a widely used and convenient diuretic, thus requires clarification.

**Methods:**

This is a prospective, single‐centered, open‐label, noninferiority phase II study. Patients with thoracic malignancies who planned to receive CDDP‐based chemotherapy were randomly assigned to receive either mannitol (arm A) or furosemide (arm B). The primary end point was set as the proportion of patients who experienced any grade of “creatinine (Cr) increased” based on the upper limit of the normal range (ULN) during the first cycle as assessed by Common Terminology Criteria for Adverse Events Version 4.0. Secondary end points were Cr increased based on the baseline value during the first cycle, Cr increased after the completion of CDDP, and the proportion of patients with phlebitis.

**Results:**

Between April 2018 and March 2022, 115 patients were enrolled and 106 were analyzed. Any grade of Cr increased based on the ULN during the first cycle was 17.3% (arm A) and 24.1% (arm B), respectively (*p* = 0.34). Therefore, the primary end point was not met. After completion of chemotherapy, any grade of Cr increased was observed in 23.1% (arm A) and 31.5% (arm B), respectively. However, the actual serum Cr level and Cr clearance during the courses were not different between the arms. Phlebitis occurred more frequently in arm A (28.8%) than arm B (16.7%).

**Conclusions:**

Mannitol should remain the standard diuresis in CDDP‐based chemotherapy assessed by conventional CTCAE grading, but furosemide can be room for consideration when assessed by actual serum Cr level and Cr clearance.

## Graphical abstract

This is the largest prospective study to compare furosemide with mannitol in the prevention of renal toxicity in CDDP‐based chemotherapy. The proportion of patients with any grade of creatinine increase during the first cycle was 17.3% in the mannitol arm and 24.1% in the furosemide arm (*p* = 0.34). Although noninferiority of furosemide to mannitol was unproven, serum creatinine levels and creatinine clearance during the courses were mostly identical.

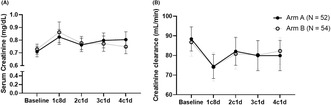

## 1 INTRODUCTION

Cisplatin (CDDP) plays an important role in the treatment of thoracic malignancies, even after the approval of novel therapeutics, such as molecular‐targeted drugs or immune‐checkpoint inhibitors.^1^, ^2^ Renal toxicity is a common issue in the management of CDDP‐induced toxicities; about 30% of patients who receive CDDP demonstrate moderate‐to‐severe renal toxicity, and this sometimes leads to CDDP dose reduction.^3^ CDDP exerts its nephrotoxic effect on the S3 segment of the proximal tubule located in the outer stripe of the outer medulla.^4^ To avoid CDDP‐induced nephrotoxicity, guidelines recommend high‐volume hydration, administration of magnesium, and forced diuresis.^5^, ^6^ Diuresis is thought to help to excrete remaining free CDDP from the renal tubules. Several data^7^, ^8^, ^9^, ^10^, ^11^ have been reported that mannitol provides renal protection during CDDP administration; currently, mannitol is the only recommended drug for this according to the National Comprehensive Cancer Network Chemotherapy Order Templates.^12^ Mannitol, which causes osmotic diuresis, increases urinary excretion of CDDP by rising osmotic pressure at the lumen of the tubules. Meanwhile, furosemide is another widely used option for diuresis although there are few reports on the nephroprotective effects.^13^ Furosemide is a loop diuretic; it blocks Na‐K‐Cl cotransporter at the ascending limb of the loop of Henle and increases urine volume by suppressing the reabsorption of sodium and water. Small randomized studies have compared the renal toxicity of mannitol with that of furosemide in 22–49 subjects,^14^, ^15^, ^16^ but there was no firm conclusion due to insufficient power to detect significant difference. Meanwhile, mannitol sometimes causes phlebitis when administered with short‐term intravenous drip infusion because of its higher osmotic pressure and a lower pH value.^17^ Furosemide is an intravenous injection; therefore, that is less likely to cause phlebitis, and also it reduces the time of administration.

This is therefore a noninferiority randomized phase II study of furosemide versus mannitol in patients with thoracic malignancies who received CDDP‐based chemotherapy using short hydration.

## 2 MATERIALS AND METHODS

### 2.1 Study design and treatment

This study was designed as a two‐arm, prospective, randomized, single‐center, open‐label phase II trial. All patients received CDDP‐based chemotherapy (≥60 mg/m^2^), so we administered neurokinin‐1 receptor antagonist, serotonin‐5‐HT3 receptor antagonist, dexamethasone, and unless contraindicated olanzapine to prevent nausea according to the ASCO guidelines.^18^

Patients were randomly assigned to the mannitol arm or the furosemide arm in a 1:1 ratio, and randomization was stratified by sex. In the mannitol arm (arm A), patients received 300 mL of 20% mannitol by intravenous drip infusion over 30 min just before CDDP. In the furosemide arm (arm B), patients received 20 mg of furosemide by intravenous injection an hour before CDDP. The chemotherapeutic agent combined with cisplatin was chosen by the physician. CDDP‐based chemotherapy was repeated every 3 or 4 weeks for up to four cycles, except where there was disease progression, unacceptable toxicity, or patients' refusal.

### 2.2 Patients

Patients had cytologically or histologically confirmed diagnosis of thoracic malignancy. They also had to fulfill the following criteria: age 20–74 years old; performance status of 0–1 on the Eastern Cooperative Oncology Group scale; no prior history of CDDP‐based chemotherapy; ability to receive CDDP ≥60 mg/m^2^; adequate renal function (serum creatinine [Cr] ≤1.2 mg/dL and a creatinine clearance [Ccr] of ≥60 mL/min). Ccr was measured by 24‐h urine collection or calculated by the Cockcroft–Gault equation. Other criteria and the study design were shown in the protocol paper of this study.^19^

### 2.3 Outcome measures

In the Common Terminology Criteria for Adverse Events (CTCAE) version 4.0, there are two definitions for “Cr increased”: One is based on the upper limit of the normal range (ULN) for serum Cr, and the other is based on each patient's baseline Cr level. Following the previous studies,^20^, ^21^ the primary end point of this study was set as the proportion of patients who had any grade of Cr increased (based on the ULN for serum Cr) during the first cycle.

Secondary end points were the proportion of patients who experienced ≥grade 2 of Cr increased (based on the ULN for serum Cr) during the first cycle, any grade and ≥grade 2 of Cr increased (based on the baseline Cr score in each patient) during the first cycle, any grade of Cr increased (based on both criteria) after the completion of chemotherapy, and the proportion of patients who had phlebitis. Evaluation and grading of phlebitis were performed by at least two medical staff.

### 2.4 Statistical analysis

Two previous prospective studies were conducted to explore the renal toxicity of mannitol with CDDP. The proportion of patients who experienced any grade of Cr increased using the CTCAE version 4.0 (based on the ULN for serum Cr) was 0%–9% during the first cycle.^20^, ^21^ Based on these studies, we assumed the proportion of patients who would have any grade of renal dysfunction in arm A would be 10%. A sample size of 51 patients in each arm provides ≥80% power to detect a noninferiority difference between the group proportions of 10% using the one‐sided binomial test with an alpha error of 0.2. Considering that approximately 10% of the patients would be censored, a total of 115 patients were required for the present study.

Efficacy analysis was performed using the full analysis set (FAS), which consisted of all randomized patients with confirmed eligibility. The primary end point was assessed by the Dunnett and Gent test using risk difference (arm B − arm A) with 80% confidence interval (CI) (one‐sided). Other outcomes were summarized with 95% CI, and comparisons between the arms were done by Fisher's exact test and Mann–Whitney *U* test. Statistical analysis was performed with R version 4.2.1 (R Foundation for Statistical Computing, Vienna, Austria).

## 3 RESULTS

### 3.1 Patient characteristics

**FIGURE 1:**
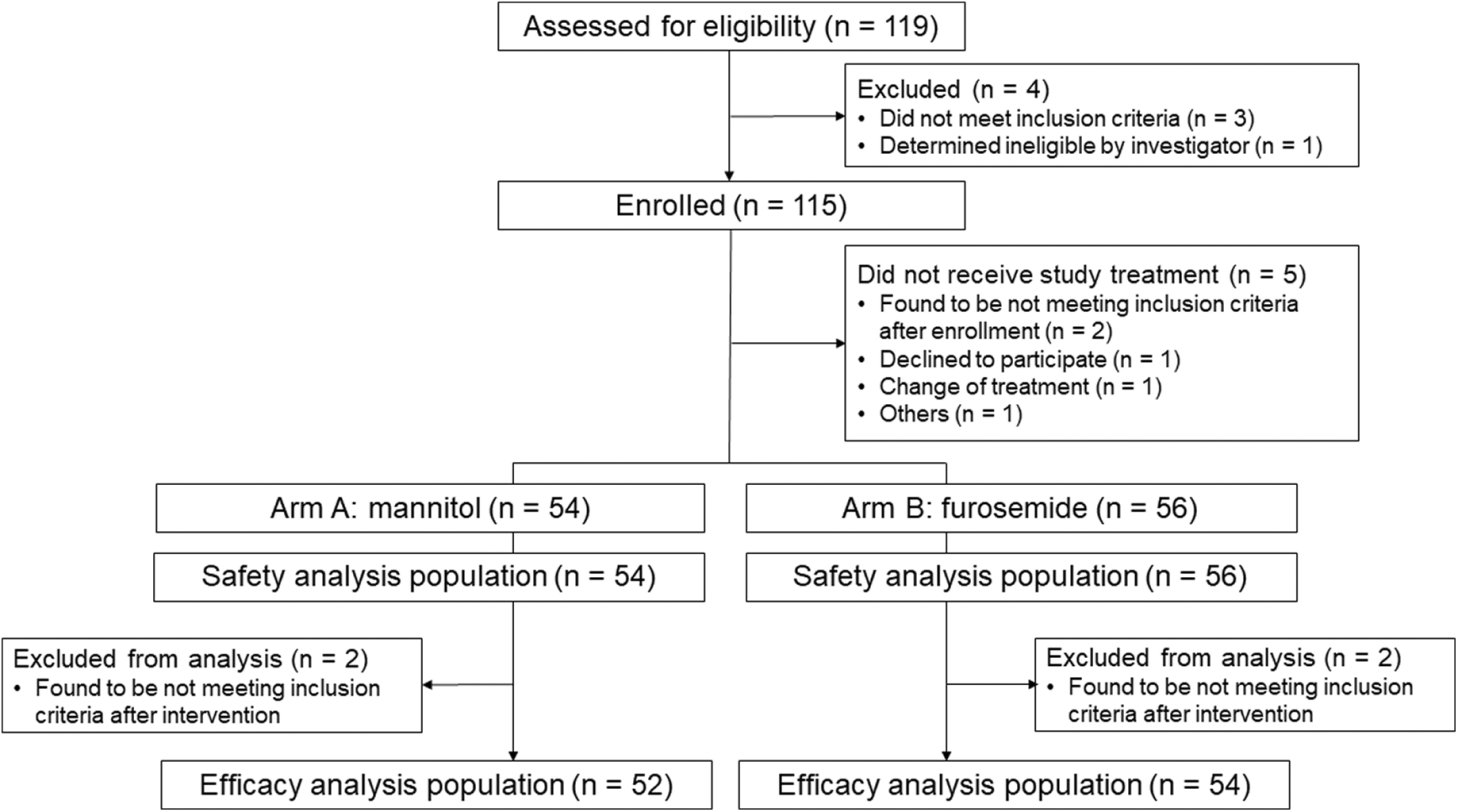
CONSORT flow diagram.

Between April 2018 and March 2022, 115 patients were enrolled, and 110 patients were eligible for the safety analysis (54 patients in arm A and 56 patients in arm B, respectively). Two patients in each arm were found not to meet inclusion criteria after treatment. The FAS population thus consisted of 106 patients (52 in arm A and 54 in arm B, respectively) (Figure 1).

Patient characteristics were well balanced between the arms (Table 1). The median age was 66.5 years and 75% were male. About 40% of the patients were stage IV and received palliative chemotherapy. About the initial dose of CDDP, in almost 65% of patients it was 80 mg/m^2^, and the median total CDDP dose was 260 mg/m^2^ in both arms, respectively. Median baseline serum Cr and Ccr were similar between the arms. The proportion of patients using nonsteroidal anti‐inflammatory drugs was 15% in both arms, and the proportion who received olanzapine to prevent nausea was 28% in arm A and 23% in arm B, respectively.

**TABLE 1:** Patient characteristics.

Characteristics	Arm A (*n* = 52)	Arm B (*n* = 54)	*p*‐Value
Age median (range)	66.5 (31–74)	66.5 (39–74)	0.69
Sex, no. (%)			
Male	39 (75)	40 (74)	1.0
Female	13 (25)	14 (26)	
Performance status, no. (%)			
0–1	52 (100)	54 (100)	1.0
≥2	0 (0)	0 (0)	
Histology, no. (%)			
Nonsmall cell lung cancer	33 (64)	37 (69)	0.84
Small cell lung cancer	11 (21)	11 (20)	
Others	8 (15)	6 (11)	
Stage, no. (%)			
IV	19 (37)	20 (37)	1.0
Others	33 (63)	34 (63)	
Treatment, no. (%)			
Adjuvant/neoadjuvant	17 (33)	17 (31)	1.0
Concurrent with radiation	14 (27)	14 (26)	
Palliative chemotherapy	21 (40)	23 (43)	
Initial dose of CDDP, no. (%)			
80 mg/m^2^	35 (67)	35 (65)	0.52
75 mg/m^2^	16 (31)	15 (28)	
60 mg/m^2^	1 (2)	4 (7)	
Total dose of CDDP			
Median (mg/m^2^, range)	260 (60–320)	260 (80–320)	0.36
Anticancer drug with CDDP, no. (%)			
Vinorelbine	24 (46)	26 (48)	0.47
Pemetrexed (+pembrolizumab)	16 (31)	14 (26)	
Etoposide (+durvalumab)	11 (21)	9 (17)	
Others	1 (2)	5 (9)	
Baseline sCr (mg/dL)			
Median (range)	0.71 (0.48–1.04)	0.75 (0.4–1.13)	0.32
Baseline Ccr (mL/min)			
Median (range)	85.8 (56.9–149.9)	81.5 (42.6–174.9)	0.46
Use of NSAIDs, no. (%)	15 (29)	15 (28)	1.0
Use of olanzapine, no. (%)	28 (54)	23 (43)	0.33

Abbreviations: Ccr, creatinine clearance (estimated by the Cockcroft–Gault equation); CDDP, cisplatin; NSAIDs, nonsteroidal anti‐inflammatory drugs; sCr, serum creatinine.

### 3.2 Outcomes

**FIGURE 2:**
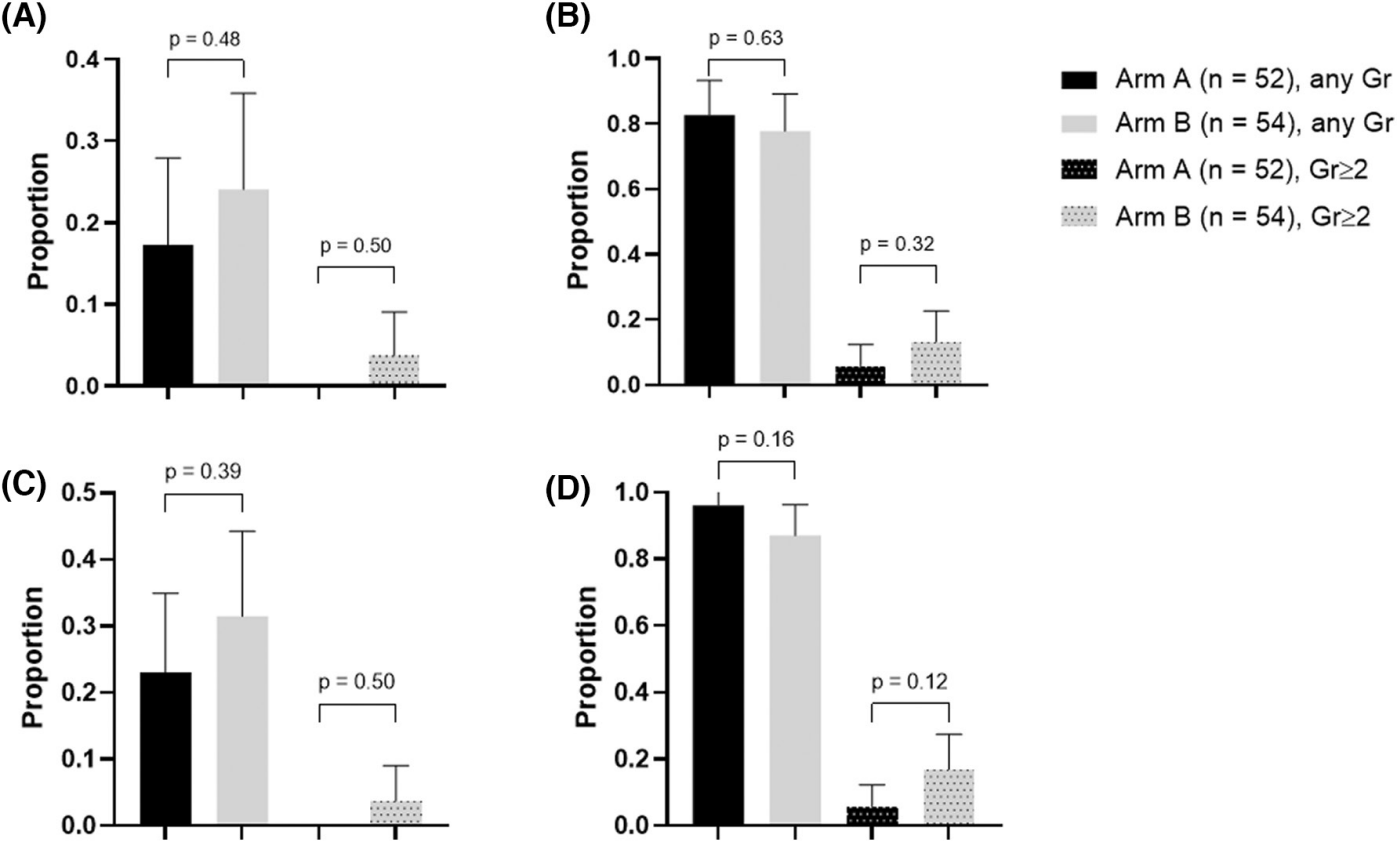
Renal toxicity. The proportion of patients who had Cr increased. (A) Based on the ULN during the first cycle, (B) based on the baseline Cr score during the first cycle, (C) based on the ULN after the completion of CDDP, (D) based on the baseline Cr score after the completion of CDDP. CDDP, cisplatin; Cr, creatinine; ULN, upper limit of the normal range.

**FIGURE 3:**
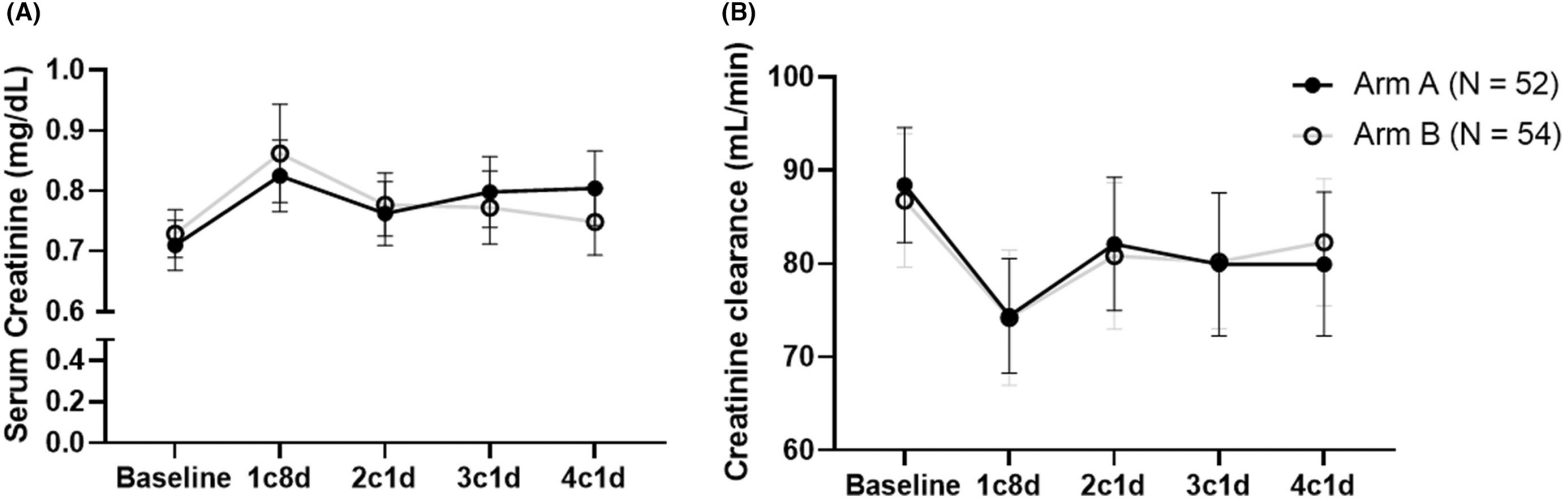
(A) Median serum Cr level and (B) Ccr during CDDP. Ccr was calculated by the Cockcroft–Gault equation. Bars represent 95% confidence intervals. Ccr, creatinine clearance; CDDP, cisplatin; Cr, creatinine.

During the first cycle, the proportion of patients who experienced any grade of Cr increased based on the ULN was 17.3% (95% CIs: 7.0–27.6) in arm A and 24.1% (95% CIs: 12.7–35.5) in arm B and yielded an absolute risk difference of 6.8 percentage points (one‐sided 80% CIs: −∞ to 13.4, *p* = 0.34 for noninferiority). To clarify, the upper boundary of the 80% CIs was over the predefined noninferiority margin of 10 percentage points indicating that the primary end point was not met. Grade 2 or higher of Cr increased based on the ULN was not observed in arm A, but was seen in 3.7% (95% CIs: −1.3 to 8.7) of patients in arm B (Figure 2A). The proportion of patients who had any grade of Cr increased based on the baseline Cr score was 82.7% (95% CIs: 72.4–93.0) in arm A and 77.8% (95% CIs: 66.7–88.9) in arm B, and ≥grade 2 of Cr increased was 5.8% (95% CIs: −0.6 to 12.1) in arm A and 13.0% (95% CIs: 4.0–21.9) in arm B, respectively (Figure 2B). After completion of CDDP, the proportion of patients who had any grade of Cr increased based on the ULN was 23.1% (95% CIs: 11.6–34.5) in arm A and 31.5% (95% CIs: 19.1–43.9) in arm B, and ≥grade 2 of Cr increased was not observed in arm A, but was seen in 3.7% (95% CIs: −1.3 to 8.7) of patients in arm B (Figure 2C). Similarly, the proportion of patients who had any grade of Cr increased based on the baseline Cr score was 96.2% (95% CIs: 90.9–101.4) in arm A and 87.0% (95% CIs: 78.1–96.0) in arm B, and ≥grade 2 of Cr increased was 5.8% (95% CIs: −0.6 to 12.1) in arm A and 16.7% (95% CIs: 6.7–26.6) in arm B, respectively (Figure 2D). Serum Cr level and Ccr during the courses were not different between the arms (Figure 3). Phlebitis tended to occur 1.7 times higher in arm A (28.8%) than in arm B (16.7%), despite the proportions of patients who received fosaprepitant and vinorelbine being similar.

### 3.3 Adverse events

There were no treatment‐related deaths. Adverse events of grade 3 or higher were febrile neutropenia (13 subjects), hyponatremia (11 subjects), anorexia (six subjects), nausea (three subjects), hypokalemia (one subject), hypercalcemia (one subject), and hearing impairment (one subject), respectively. The safety profile was generally similar between the arms (Table 2).

**TABLE 2:** Adverse events.

	Arm A (*n* = 54)		Arm B (*n* = 56)		*p*‐Value
	Any grade, no. (%)	Grade3/4, no. (%)	Any grade, no. (%)	Grade3/4, no. (%)	
Hyponatremia	43 (80)	6 (11)	45 (80)	5 (9)	1.0
Anorexia	25 (46)	4 (7)	29 (52)	2 (4)	0.57
Nausea	21 (39)	1 (2)	23 (41)	2 (4)	0.85
Constipation	17 (31)	0	26 (46)	0	0.12
Hypercalcemia	8 (15)	0	6 (11)	1 (2)	0.58
Febrile neutropenia	6 (11)	6 (11)	7 (13)	7 (13)	1.0
Hypokalemia	5 (9)	0	7 (13)	1 (2)	0.76
Diarrhea	3 (6)	0	3 (5)	0	1.0
Vomiting	1 (2)	0	2 (4)	0	1.0
Hearing impairment	0	0	1 (2)	1 (2)	1.0

## 4 DISCUSSION

This randomized controlled phase II trial was conducted to evaluate the renal preventive efficacy of furosemide compared with mannitol in patients with thoracic malignancies receiving CDDP‐based chemotherapy. Several previous studies^14^, ^15^, ^16^ comparing mannitol and furosemide have been published, but results were derived from limited number of subjects (22–49 patients). We consider that this study is the first prospective study that enrolled a sufficient number (≥100) of patients even though this has the statistical drawbacks (relatively higher *α* and *β* errors). Regarding statistical considerations, more strict setting was desirable; however, very limited space was left to conduct this type of research because many clinical trials of novel anticancer drugs have been ongoing. Taking this situation into account, we believe that the statistical plan is barely acceptable. Although the primary end point was not met, the proportional difference in Cr increased between the two arms in the first cycle was within clinically acceptable ranges. More importantly, the actual serum Cr levels and Ccr during courses were generally similar between the arms (Figure 3). Thus, the result can provide relevant insight that furosemide showed almost identical serum Cr and Ccr changes despite CTCAE‐based nephrotoxicity was slightly different. A retrospective study that examined the nephroprotective effects of mannitol and furosemide in cervical cancer patients who received CDDP^22^ also reported similar the actual changes in serum Cr levels and Ccr in both arms. CTCAE grading is just a numerical change of serum Cr and does not consider the subject's sex, body weight, or age. That is, Cr increased in CTCAE sometimes does not reflect the actual renal toxicity. This gap may have a clinically debatable issue. We adopted CTCAE grading as the primary end point on the basis of a previous study; however, future trials to assess CDDP‐induced renal toxicity should pay more attention to this point.

Another relevant point in this study is the assessment of phlebitis. As previously mentioned, mannitol sometimes causes phlebitis, and such an adverse event affects patients' quality of life. Although the difference was not statistically different due to the small sample size, the incidence of phlebitis was 1.7 times higher in the mannitol arm. Furthermore, considering the convenience of furosemide administration, furosemide also may be room for consideration in CDDP‐based chemotherapy with similar serum Cr changes, less skin toxicity, and convenience.

This study has several limitations. First, a phase II, noninferiority study is insufficient to reach a rigid conclusion. However, to conduct the phase III trial in the same design is unrealistic in terms of patient resources. Second, this is a single‐center, open‐label study. Although a multicenter organization would have made it possible to conduct a phase III trial, this limitation was difficult to overcome because some researchers were not interested in spending their efforts on this topic rather than clinical trials with the novel anticancer agents, even though this is the unresolved clinical question. Finally, regarding the assessment of renal function, this study has used Ccr, which was measured by 24‐h urine collection or calculated by the Cockcroft–Gault equation according to previous studies. However, they tend to overestimate the actual glomerular filtration rate (GFR); thus, it may be beneficial to calculate using the chronic kidney disease‐epidemiology collaboration (CKD‐EPI) equation or other estimated GFR (eGFR) equation for Japanese.

In conclusion, our result demonstrates that while mannitol is the standard diuresis in CDDP‐based chemotherapy by conventional CTCAE grading, furosemide also can have room for consideration when assessed by actual Cr level and Cr clearance.

## AUTHOR CONTRIBUTIONS

**Eriko Murakami:** Conceptualization (lead); data curation (lead); formal analysis (lead); investigation (lead); writing – original draft (lead); writing – review and editing (lead). **Hiroaki Akamatsu:** Conceptualization (lead); investigation (lead); supervision (lead); writing – original draft (lead); writing – review and editing (lead). **Shunsuke Teraoka:** Conceptualization (lead); investigation (lead); writing – review and editing (equal). **Toshiaki Takakura:** Investigation (equal); writing – review and editing (equal). **Eri Takase:** Investigation (equal); writing – review and editing (equal). **Masanori Tanaka:** Investigation (equal); writing – review and editing (equal). **Takahiro Kaki:** Investigation (equal); writing – review and editing (equal). **Yuhei Harutani:** Investigation (equal); writing – review and editing (equal). **Katsuyuki Furuta:** Investigation (equal); writing – review and editing (equal). **Takeya Sugimoto:** Investigation (equal); writing – review and editing (equal). **Ryota Shibaki:** Investigation (equal); writing – review and editing (equal). **Daichi Fujimoto:** Investigation (equal); writing – review and editing (equal). **Atsushi Hayata:** Investigation (equal); writing – review and editing (equal). **Yuichi Ozawa:** Investigation (equal); writing – review and editing (equal). **Masanori Nakanishi:** Investigation (equal); writing – review and editing (equal). **Yasuhiro Koh:** Investigation (equal); writing – review and editing (equal). **Toshio Shimokawa:** Formal analysis (lead); writing – review and editing (equal). **Nobuyuki Yamamoto:** Project administration (lead); supervision (lead); writing – review and editing (lead).

## CONFLICT OF INTEREST STATEMENT

All authors have no conflicts of interest to declare.

## ETHICS STATEMENT

All procedures were performed in accordance with the Declaration of Helsinki, and the protocol and amendments were approved by the Wakayama Medical University Certified Review Board (CRB5180004). All patients provided written informed consent before participation in the study. This study was registered at the University Hospital Medical Information Network Clinical Trials Registry (UMIN000031910) and at the Japan Registry of Clinical Trials (jRCTs051180015).

## Data Availability

The datasets are available from the corresponding author on reasonable request.
